# A New SCAE-MT Classification Model for Hyperspectral Remote Sensing Images

**DOI:** 10.3390/s22228881

**Published:** 2022-11-17

**Authors:** Huayue Chen, Ye Chen, Qiuyue Wang, Tao Chen, Huimin Zhao

**Affiliations:** 1 School of Computer Science, China West Normal University, Nanchong 637002, China; 2 School of Electronic Information and Automation, Civil Aviation University of China, Tianjin 300300, China; 3 Traction Power State Key Laboratory, Southwest Jiaotong University, Chengdu 610031, China

**Keywords:** stacked convolutional autoencoder network, transfer learning, feature extraction, small samples, HRSI

## Abstract

Hyperspectral remote sensing images (HRSI) have the characteristics of foreign objects with the same spectrum. As it is difficult to label samples manually, the hyperspectral remote sensing images are understood to be typical “small sample” datasets. Deep neural networks can effectively extract the deep features from the HRSI, but the classification accuracy mainly depends on the training label samples. Therefore, the stacked convolutional autoencoder network and transfer learning strategy are employed in order to design a new stacked convolutional autoencoder network model transfer (SCAE-MT) for the purposes of classifying the HRSI in this paper. In the proposed classification method, the stacked convolutional au-to-encoding network is employed in order to effectively extract the deep features from the HRSI. Then, the transfer learning strategy is applied to design a stacked convolutional autoencoder network model transfer under the small and limited training samples. The SCAE-MT model is used to propose a new HRSI classification method in order to solve the small samples of the HRSI. In this study, in order to prove the effectiveness of the proposed classification method, two HRSI datasets were chosen. In order to verify the effectiveness of the methods, the overall classification accuracy (OA) of the convolutional self-coding network classification method (CAE), the stack convolutional self-coding network classification method (SCAE), and the SCAE-MT method under 5%, 10%, and 15% training sets are calculated. When compared with the CAE and SCAE models in 5%, 10%, and 15% training datasets, the overall accuracy (OA) of the SCAE-MT method was improved by 2.71%, 3.33%, and 3.07% (on average), respectively. The SCAE-MT method is, thus, clearly superior to the other methods and also shows a good classification performance.

## 1. Introduction

Hyperspectral remote sensing images (HRSI) have the characteristics of “homospectral foreign objects” and “homospectral foreign objects”. In order to accurately identify and classify the ground objects, the deep features of the HRSI need to be extracted. These deep features can fully represent the original ground objects, such as spectral features, spatial features, and local correlation features, in addition to the shallow and middle-level features such as the shape, contour, and texture of the images [1,2,3,4]. In recent years, an autoencoder network has obtained good results in the use of the deep feature extraction of HRSI via the decoding and coding of images, feature mapping, and image reconstruction in multi-layer networks [5,6,7]. Zhang et al. [8] presented an unsupervised feature learning method using a RAE (recursive autoencoders) network. However, the recursive structure will occupy a lot of space and has low efficiency. If the recursive structure is too deep, it will cause stack overflow and program crash. Shi and Pun [9] proposed a recursive neural network that was based on a stack autoencoder in order to learn high-order multiscale spectral spatial features. However, this was also troubled by efficiency and easy “overfitting”. Mei et al. [10] proposed a spectral and spatial feature extraction method based on a three-dimensional convolutional autoencoder (3D-CAE) in order to extract the deep features of the HRSI. However, in the 3D operation of the deep neural network, the training process is complex, i.e., the “small sample” and efficiency problems are still the bottleneck of the classification task. Zhao et al. [11] proposed a simplified three-dimensional convolutional self-encoder (S3D-CAE) for the purposes of extracting the spatial features of deep spectrum from the dual temporal images without prior information. Although the spectral redundancy is reduced and the data processing speed is improved, it is easy for “salt noise” to appear when the classification task is completed. Zhang et al. [12] designed a bi-directional self-encoder, which reconstructed hyperspectral data and lidar data together, as well as sent the fused data to a dual-branch CNN for final classification, which improved the classification accuracy. However, the model training still requires one to consider many parameters.

The autoencoder network can effectively extract the deep features of the HRSI through the layer-by-layer training of the deep neural network structure [13,14,15]. However, when the model is trained, the scale of involved parameters in the training is large, the computational complexity is high, and the efficiency is low. In the full connected network, it is also easy to ignore local dependencies in the domain space. On the other hand, it is difficult to manually label samples of the HRSI data, which are examples of typical “small sample” data. Insufficient training samples and labeled samples will further lead to difficult and unstable training parameters of the classification model; further, the insufficient space-spectral feature space will also affect classification accuracy [16,17,18,19]. Dai et al. [20] combined a residual convolutional neural network (RESNET) with an ensemble learning (EL) strategy in order to establish discriminant image representation to complete the classification task. Consequently, they obtained good classification results. However, the classification task was completed via supervised learning, which has a requirement to be based on a certain number of labeled samples. Yan et al. [21] introduced the semi-supervised learning into the generative adversarial network (GAN) and introduced the aliasing data enhancement method in the classification model, which increased the available marker data for classification and improved the classification accuracy. However, the model is prone to “over-fitting”. Miao et al. [22] proposed a semi-supervised representation-consistent Siamese network (SS-RCSN) in order to reduce the difference between labeled and unlabeled data to a certain extent, which, thus, prevents the model from “overfitting”. However, the intra-class diversity and inter-class similarity make it easy to generate “salt and pepper noise”, which affects the accuracy of classification. In addition, some other methods are also proposed to deal with images and other problems [23,24,25,26,27,28,29,30,31,32,33,34].

On basis of the idea of semi-supervised learning, the insufficient labeled sample data can be solved to a certain extent [35,36,37,38,39,40,41,42]. However, for the HRSI data—due to the characteristics of the spatial structure, scale size, spectral confusion, correction error, etc.—the real labels of the feature data and the learned pseudo labels from the training data are easy to mislabel and are inconsistent. Further, if this part of the unlabeled sample data is forcibly added to the training dataset of labeled samples, it will easily affect the classification accuracy. He et al. [43] presented a HRSI classification method based on heterogeneous transfer learning. However, the “fine-tuning” process of the structure of two heterogeneous datasets during transfer is complicated. Chen et al. [44] proposed a semi-supervised dual-dictionary nonnegative matrix factorization (SSDDNMF) heterogeneous transfer learning algorithm that projects source domain features and target domain features. In the dimensional subspace, the difference between feature spaces is eliminated, but its key features are easily lost when the features are shared. Additionally, the determination of size difference has a greater impact on the classification accuracy. Qu et al. [45] reconciled the gap between the source domain dataset and the target domain dataset by projecting the HRSI data of the source domain and the target domain into the shared abundance space, as based on their physical characteristics. Yang et al. [46] designed a hierarchical depth neural network in order to realize the shallow feature transfer and depth feature classification, as well as to reduce the demand for source domain data.

To summarize, the autoencoder network is an unsupervised learning model. When the hyperspectral remote sensing image features are learned, it can extract the spatial spectrum features, but it will lose the spatial information of the original images in the training process. On the other hand, the deep network model needs a large number of labeled samples in order to participate in the training, while the hyperspectral remote sensing image data is an example of typical “small sample” data and the lack of training samples is easy to make the model produce an “over fitting” result. The transfer strategy is introduced in order to solve the training problem of “small sample” data to a certain extent. However, the different acquisition conditions of the HRSI usually leads to spectral changes, while the training and test data are usually located in different domains, and the data distribution changes not only with time and space, but also with different situations. It is sensitive to aim of reconciling the gap, but its classification robustness is poor. In order to solve these problems, a stacked convolutional autoencoder network model is employed in order to extract the deep features from the HRSI. As the different training datasets have different distribution spaces during model training, and the learning model cannot use the same data distribution to treat new training data, a transfer learning strategy is introduced in order to modify the learning model to be more robust. The model parameters, structure, and other knowledge transfers are trained; further, the learning model is continuously fine-tuned during the transfer process, such that the trained model can be adjusted before the new data set is employed in order to solve the model sensitivity and poor classification robustness under “small samples” readings. It is, therefore, easy to obtain the unsatisfactory classification accuracy caused by “over-fitting”. Moreover, the classification effectiveness and accuracy of the HRSI are improved.

The innovations and main work of this paper are described as follows.

- A new stacked convolutional autoencoder network model transfer (SCAE-MT) is developed;
- The stacked convolutional auto-encoding network is used to effectively extract the deep features of the HRSI;
- The transfer learning strategy is employed in order to develop a SCAE network model transfer under small and limited training samples;
- The SCAE-MT is used to propose a new HRSI classification method in order to solve the small samples that can be found in the HRSI.

## 2. Image Classification with SCAE-MT

### 2.1. The Idea of Image Classification

In order to accurately identify and classify the ground objects, when the massive high-dimensional hyperspectral remote sensing image data is used, it is necessary to extract image features that can fully represent the original ground objects [47,48,49,50]. Therefore, in this paper, the stack convolution auto-coding network is employed in order to extract the deep features of hyperspectral remote sensing images. All convolution cores, weights, and offsets in the deep neural network are trained through a deep iteration process. Further, in the process of training, the optimal convolution kernel parameter weight w and bias b are obtained by repeatedly alternating a forward propagation and backward error return. Clearly, when the stack convolutional auto-coding network is to be iteratively trained, sufficient marker samples are required in order to participate in the training of the deep neural network. The marker samples involved in the training are greater, the accuracy of acquiring the deep features is higher, and the recognition accuracy of ground objects are also higher. However, the characteristics of the HRSI data determine that it is difficult to label samples [51,52,53,54]. Therefore, if the HRSI dataset is simply trained by a deep neural network—which is based on a stack convolution self-encoder—due to the fact that the involved label samples in training are limited, then it is easy to produce the phenomenon of “over fitting”.

In order to solve this problem, a transfer learning strategy was introduced when the stack convolution self-encoder model was trained. As the HRSI datasets usually have different distributions of their source domain datasets and target domain datasets, the model-based transfer learning is adopted. Firstly, the source domain datasets are pre-trained, and the source domain datasets and target domain datasets share some general prior knowledge on the model. The HRSI data sets from different scenes collected by the same sensor are common to the image itself in terms of shape, corner, and other characteristics. Though the source domain data set is pre-trained, the transfer knowledge and code for it to model parameters will participate in model fine-tuning; in addition, it will help complete the classification task of target domain datasets via the adjusting of previous prior knowledge and pre-trained models.

### 2.2. Processes of Implementation

For the purposes of the HRSI classification, the HRSI datasets from different scenes collected by the same sensor are common to the image itself, such as the shape and corner. In this paper, the transfer learning strategy was used to transfer these common features from the source dataset to the target dataset. Additionally, the transferred knowledge is applied to the model prior to knowledge acquisition, model parameter training, and model architecture of the target dataset. For the top layer of the target scene dataset, the deep neural network is used to extract the deep features of the images and classify the target scene data [55,56,57]. The specific implementation steps are described as follows.

Step 1: Deep feature extraction

The HRSI data is composed of two-dimensional spatial information and one-dimensional spectral information. It is a three-dimensional data. Compared with ordinary remote sensing images, it contains more abundant spectral information and spatial information. For the HRSI data, the invariance and limited robustness of it shallow features are difficult to cope with in regard to the high intra-class variability and low inter-class variability. The auto-encoding network cascades multiple layers of features and extracts higher-level features hierarchically. For the HRSI data, it can effectively combine its spectral features and spatial features, which is beneficial in order to improve the classification accuracy of ground and object. However, during the training of the self-encoding network model, because the HRSI data is usually represented as a vector, the full connected layer will lose spatial information. With the increase in the number of neurons in the hidden layer, the parameter size of the weight matrix will also increase sharply. Many parameters will lead to difficult and inefficient training; further, the model is prone to “over-fitting”. Therefore, the convolutional layers with local connection and weight sharing characteristics are used to replace the convolutional autoencoder network with a full connected layer of simple autoencoder networks for the purposes of deep feature extraction of the HRSI [58,59,60]. When the convolutional self-encoding network is trained—due to the fact that the neurons in each layer are arranged in three dimensions of width, height, and depth—the neurons in the layer are only connected to a small area in the previous layer, instead of it taking a full connection. The involved parameters in training are relatively small, which reduces the computational complexity and solves the problem of model “over-fitting” to a certain extent. On the other hand, the local connection and weight sharing of the convolutional layer and the three-dimensional arrangement of the feature vectors enable the convolutional auto-encoding neural network to better preserve the local spatial information of the data, as well as capture a specific type of input data through a convolution kernel. The local features of the HRSI can be extracted by using multiple different convolutions in order to extract multi-level features.

**Figure 1:**
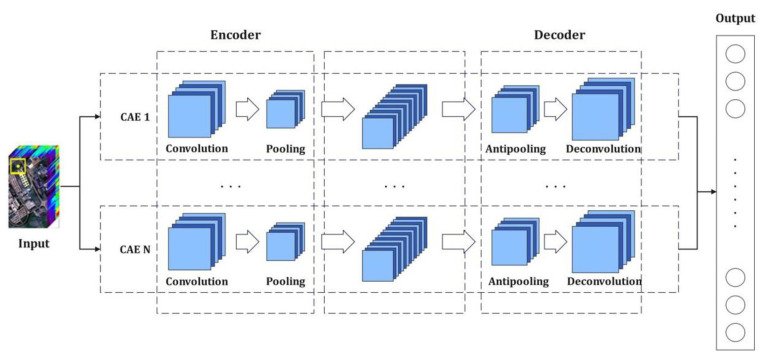
The stacked convolutional autoencoder network model.

In order to strengthen the ability of the convolutional autoencoder network to better extract the deep features of the HRSI, the classification accuracy requires further improvement. The convolutional autoencoder networks in a stack manner are organized to construct an unsupervised deep neural network for the purposes of extracting the deep features of the HRSI through stacked convolutional autoencoder networks [61,62,63,64,65,66]. The stacked convolutional autoencoder network utilizes the characteristics of local connections and the weight sharing of convolutional layers; further, it is trained layer-by-layer from low layers to high layers. While the computational complexity of the training model is reduced, the features of each layer are fused and reconstructed in order to generate fusion deep-level feature representation for spectral and spatial features of the HRSI. The stacked convolutional self-encoding network model is shown in Figure 1.

Suppose the stacked convolutional auto-encoding network is composed of *n* convolutional auto-encoding networks and that the input feature is $X=\left\{x_{i}|\left(i=1,\;2,\cdots m\right)\right\}$. Then the features of the output of the l(l=1, 2,⋯n) convolutional layer are expressed as follows:

(1) $$z^{l}=\sigma \left(x_{i}⊗w_{l}^{k\times k}+b^{l}\right)$$

(2) $$Y=\sigma \left(\overset{l}{\underset{d=1}{\sum }}x_{i}^{d}⊗w_{d}^{k\times k}+b_{d}^{l}\right)$$

Among these, $z^{l}$ is the feature map of the *l* layer, *Y* is the superposition of all feature maps, $w_{l}^{k\times k}$ is the size of k×k the convolution kernel, $b^{l}$ is the bias, and ⊗ represents the convolution operation. As the image has a local correlation, the input feature vector can extract the features that have a local correlation through the convolution operation with the convolution kernel. After the convolution operation, the pooling layer is used for the purposes of pooling in order to reduce the amount of training parameters and to also prevent over-fitting. In this paper, mean pooling is used; further, σ(·) is a nonlinear differentiable activation function, and the ReLU function with fast convergence speed and simple calculation is employed.

In order to reduce the number of parameters and computational complexity, as well as to improve the training efficiency, the high-dimensional features are converted into low-dimensional features through operating a series of convolution pooling; then, the merged smaller images are unsampled into the original image dimension. The low-dimensional feature vectors are used to reconstruct the images, that is, the low-dimensional features are mapped to high-dimensional features. After each convolution layer passes through the activation function, the feature map $z^{l}$ is obtained, and the transposition of the corresponding convolution kernel is used to perform the convolution operation by feature reconstruction. The specific expression is described as follows:

(3) $$Y=\sigma \left(\overset{l}{\underset{i=1}{\sum }}z^{l}⊗\tilde{w_{l}^{k\times k}}+b^{l}\right)$$

Among this, the $w_{l}^{k\times k}$ transpose matrix is of $\tilde{w_{l}^{k\times k}}$, and the reverse $w_{l}^{k\times k}$ mapping of low-dimensional features to high-dimensional features is realized by transposing.

The parameters in the stacked convolutional autoencoder network are the weights and biases in the convolutional layer, and the backpropagation (BP) algorithm is used in order to update the parameters. The updating process of the parameters via the use of the BP algorithm is divided into forward propagation and back propagation. According to the initialization weight *w* and bias *b* given by the input sample, the square error is used as the loss function, which is then used to calculate the error value δ between the output value *y* and the actual value *x*.

(4) $$\delta =\frac{1}{2}\overset{l}{\underset{i=1}{\sum }}{\left(y_{i}-x_{i}\right)}^{2}$$

The above is a forward calculation process. If the error δ is within the threshold range at this time, it stops updating the weights *w* and bias *b*. On the contrary, if the error δ value is not within the threshold range, then the error is returned for back propagation. During the process of backpropagation, the gradient descent method is used to solve the weight *w* and bias *b*, such that the minimum error reaches the threshold range. In the stacked convolutional auto-encoding process, the gradient is backpropagated through the error value δ of each layer. Therefore, during backpropagation, the rule is employed in order to calculate the gradient of the convolution layer parameters. Further, the loss function is used to calculate the gradient of the *l* layer. Partial derivatives for weights $w^{l}$ and bias $b^{l}$ are described as follows.

(5) $$\frac{\partial \delta }{\partial w^{l}}=\frac{\partial \delta }{\partial z^{l}}⊗x^{l-1}$$

(6) $$\frac{\partial \delta }{\partial b^{l}}=\frac{\partial \delta }{\partial z^{l}}⊗x^{l-1}$$

In the deep feature extraction of the HRSI, the model is pretrained by using stacked convolutional autoencoder networks. Further, in the pre-training process, the first-order features are obtained from the bottom layer; that is, the first layer is used as the input of the second layer. When compared with the first-order features of the first layer, the second-order features including the first-order feature pattern of the next layer will be learned in the second layer. By the use of analogy and training layer-by-layer, the trestle convolution self-coding network will eventually learn the higher-level features of the HRSI data. Compared with the convolutional autoencoder network, the stack-type convolutional autoencoder network is stacked in a stacking manner in the training phase, such that the spatial correlation features of the underlying features are retained in the upper-layer feature map. The local correlation of features within the domain is well preserved. This solves the problem that a single convolutional autoencoder network easily loses, i.e., the spatial correlation features of the domain.

Step 2: Classification based on SCAE-MT

**Figure 2:**
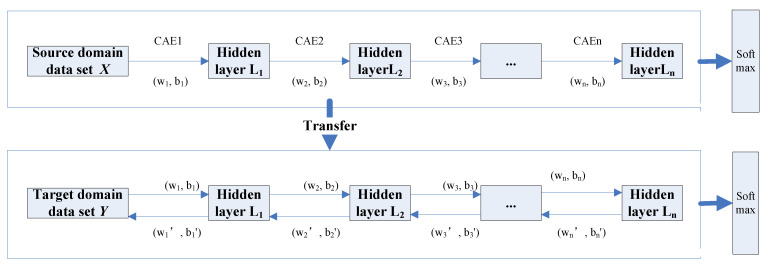
The classification framework based on the stacked convolutional autoencoder.

The stacked convolutional autoencoder network is used as the training and base model for transfer learning. The pre-training is performed on the source-domain HRSI dataset. At this time, the parameter weights $w_{i}$ and biases $b_{i}$ of the stacked convolutional auto-encoding network are randomly generated during initialization. After the pre-training of the source domain dataset is completed, the model parameters obtained by the pre-training network are transferred by the transfer strategy of sharing model structure and parameters. The weights $w_{i}^{\prime }$ and biases $b_{i}^{\prime }$ are considered as initialization parameters in regard to the target domain training dataset model for the purposes of training the target domain dataset. In regard to the ground feature classification, in order to improve the dataset due to limited sample labels, the model parameters of the weights and biases for the target domain dataset are obtained by fine-tuning within the process of the training. Additionally, the transfer strategy is used to complete the feature vector construction of the “small sample” dataset, as well as to reduce the impact of insufficient training samples on the classification performance. Moreover, the classification framework is transferred based on the stacked convolutional autoencoder model in Figure 2.

In the transfer process, as based on the stacked convolutional auto-encoding network model, the average value of the softmax activation function of all source samples of the category ${\mathrm{class}}_{n}$ and the ${\mathrm{class}}_{n}$ weak label t are calculated. Further, that which can be extracted then its average value $t^{\left(n\right)}$ is used here. The cross-entropy loss of the source domain dataset and the target domain dataset can be defined as follows:

(7) $$\zeta =\frac{1}{n}\underset{i}{\sum }\zeta_{i}=\frac{1}{n}\underset{i}{\sum }-\sum_{c=1}^{n}x_{ic}\log t_{ic}^{n}$$

When the trained model on the source domain data set is used as prior knowledge in order to train the model of the target domain dataset, whether the target domain sample belongs to a category $c_{i}$ or is calculated by defining an excitation function, then the excitation function is defined as follows:

(8) $$f=\left(y_{i},c_{i}\right)=t^{\left(n\right)}\cdot \bar{\theta }\left(y_{i},c_{i}\right)=t^{\left(0\right)}\cdot \theta^{0}\left(x,y\right)+\sum_{z=1}^{covn}t^{\left(c_{i},z\right)}\cdot \theta^{\left(c_{i},z\right)}$$

where $\theta^{\left(\cdot \right)}$ is the feature mapping function, and *covn* is the number of layers.

The steps of the fine-tuning are described as follows:

Step 1. Train a stack convolutional auto-coding model on the source domain dataset as the source model.

Step 2. The target domain dataset copies the model structures and parameters on the source domain model except in regard to the output layer. Then the stack convolutional auto-coding network model of the target domain dataset is established.

Step 3. Add an output layer whose output size is the number of target domain dataset categories to the target domain model, as well as randomly initialize the model parameters of this layer.

Step 4. The target model is trained on the target domain dataset. At this time, the structure and weight parameters of all previous layers (i.e., those that do not participate in training, which is to say do not back propagate) are frozen. Then, a smaller learning rate and the gradient descent method are used in order to retrain the output layer.

## 3. Experiment Results and Analysis

In order to verify the effectiveness of the proposed classification method based on the SCAE-MT, the Indian Pines dataset and the Salinas dataset are selected here. They are the two commonly hyperspectral remote sensing image datasets by the same sensor in different areas and they are used as the experimental datasets.

### 3.1. Experimental Datasets

Both the Indian Pines and the Salinas datasets were acquired by the airborne visual/infrared imaging spectrometer AVIRIS, the basic information of which is shown in Table 1. The Indian Pines dataset is an image of an Indian pine tree in Indiana, USA; further, the size of 145 × 145 is then intercepted and marked as a dataset for the HRSI classification, which is one of the most commonly used datasets. The data contains a total of 21,025 pixels—excluding a large number of background pixels—and the remaining pixels containing ground objects are a total of 10,249, which includes 16 types of ground objects and 200 valid bands. The statistics of these ground object categories are shown in Table 2. For the Salinas dataset, it originates from the Salinas Valley in California, USA. Compared with the Indian Pines dataset, the Salinas dataset is larger, has more samples, and has a higher spatial resolution of 3.7 m. The image size is 512 × 217, as such it contains 111,104 pixels in total, of which 56,975 are background pixels. Additionally, there are 54,129 pixels that can be used for the purposes of classification, including 16 types of ground objects and 204 valid bands. The category statistics of this are shown in Table 2.

### 3.2. Experiment Environment and Parameter Settings

The experiment environment was configured as follows: thinkstationp920 was the workstation, the CPU@2 *Xeon silver 4210, memory (RAM) was 128G, the graphics card was an NVIDIA Titan RTX Titan (video memory 24G), and the programming language used was Matlab R2018a.

During the experiment, the kernel size of the convolution self-coding network was the ownership value matrix of the full connection layer that was initialized according to a Gaussian random distribution; further, the standard deviation was set to 0.05. Additionally, the mean value was set to 0. The bias initialization was set to 0, the mean pooling method was adopted, and the activation function was the ReLu function. The learning rate in the standard random gradient descent method was fixed to 0.0001, the momentum was set to 0.9, and the batch size (bottom, 2012) was set to 128. The maximum number of iterations was 80.

### 3.3. Experimental Results and Analysis

**Figure 3:**
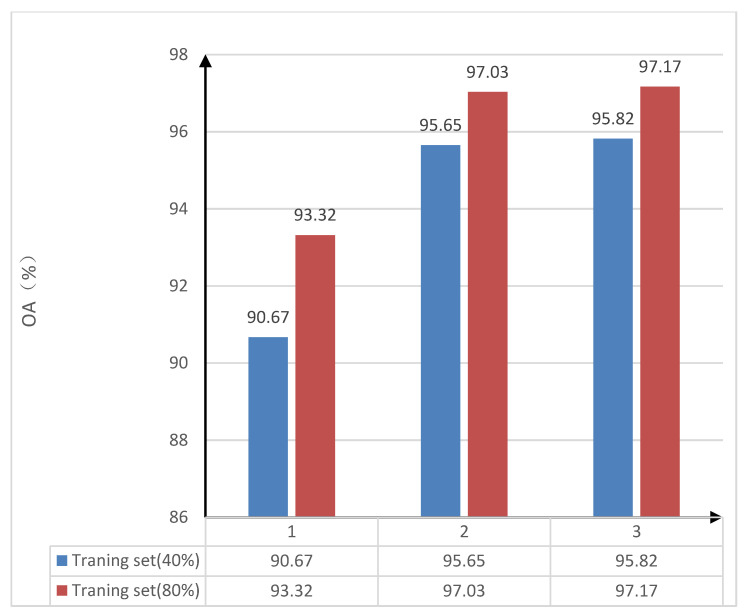
The classification effect of different convolutional self-coding networks.

In order to verify the learning ability of the trestle convolutional self-coding network, an experimental comparison of the sensitivity of the number of stacked convolutional self-coding networks was executed on the classification performance in the network structure. The 40% and 80% of the Indian Pines dataset were randomly selected as training samples according to prior experiences and the literature; the rest was the test data set. The training data set was used to train the model, and the test data set was used to verify the effectiveness of the model, which were trained under the model with the number of convolutional self-coding networks of 1, 2, and 3, respectively. Additionally, the overall accuracy (OA) of the classification was calculated. The overall accuracy, i.e., the OA values of classification under different convolutional self-coding networks and also under different training samples are shown in Table 3. Table 3 shows the overall classification accuracy of the Indian Pines dataset by randomly selecting 40% and 80% of the samples as the training set, then training and calculating when the number of convolutional self-encoders is 1, 2, 3. The comparative histogram of the sensitivity of the number of convolutional self-coding networks to classification performance in the trestle convolutional self-coding model is shown in Figure 3. In order to clearly compare the impact of different number of self-encoders on the classification accuracy, the column chart is drawn to show that in the stack type convolutional self-coding network, with the increase in the number of convolutional auto-encoders, the overall classification accuracy will increase. A reference is provided for subsequent experiments to help determine the number of convolutional auto- encoders.

From Table 3 and Figure 3, the overall accuracy (OA) of classification is the lowest when there is only one convolutional self-coding network in the trestle convolutional self-coding network that is under 40% or 80% in the training sample sets. When the number of convolutional self-coding networks in the trestle convolutional self-coding model structure increases, the overall accuracy (OA) of classification will also increase. However, it can be seen that when the number of convolutional self-coding networks in the model result is three, then the overall accuracy of classification is improved by 0.17% and 0.14%, respectively, when under 40% and 80% in regard to the training sample sets by comparing with the case of only two networks. The increasing number of convolutional self-coding networks has little impact on the classification accuracy. Considering the running cost of adding one convolutional self-coding network, as well as in balancing the time-space efficiency and classification accuracy (OA), two convolutional self-coding networks are selected in order to participate in the training of the trestle convolutional self-coding model.

**Figure 4:**
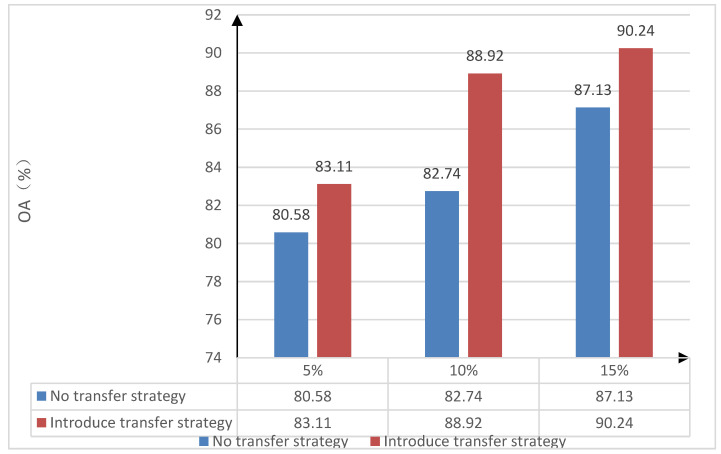
The impact of the transfer strategy on the classification accuracy.

In order to verify the effectiveness of the transfer learning strategy in the proposed classification method—assuming that the Indian Pines dataset has sufficient training samples, which is considered as the source domain dataset—and the Salinas data set acquired by the same sensor in different regions is the target data set, then the following steps should be taken. Firstly, the effectiveness of the model transfer strategy is verified. The 80% of the samples on the Indian pines data set is selected for the purposes of pre-training on the trestle convolutional self-coding network model. Further, the Indian pines data set with sufficient samples was selected as the source domain dataset. Then, the “small sample” data on the Salinas data set are randomly selected as the training set, and the 5% on the Salinas data set, in turn, are randomly selected. The 10% and 15% of the samples were used as training sample sets; further, experiments were conducted on the influence of a non-transfer learning strategy and the introduction of a transfer learning strategy on the overall classification accuracy of “small sample” data sets. Under the no transfer learning strategy, the “small sample” training sets of 5%, 10%, and 15% on the Salinas data set were trained, respectively. In addition, the overall classification accuracy (OA) was obtained after the random initialization parameters were trained. After the transfer learning strategy was used, the model-based transfer learning strategy was introduced when the target domain dataset Salinas 5%, 10% and 15% of the “small sample” training sets are trained. The parameters of all layers in the structure of the pre-training model are used as the initial parameters for training under different numbers of small samples in the Salinas dataset of the target domain, and the gradient descent method was used to fine tune them to obtain the overall classification accuracy (OA) under each proportion of the training sample set. The overall classification accuracy (OA) of the “small sample” training set without a transfer strategy, as well as after the introduction of a transfer strategy on the Salinas dataset, are both shown in Table 4. The comparative histogram of the overall classification accuracy (OA) of the three ratios without a transfer strategy and after the introduction of a transfer strategy are shown in Figure 4.

From Table 4 and Figure 4, after the transfer strategy is introduced, the overall accuracy of the target domain in regard to the Salinas dataset, under the small-sample training set of 5%, 10%, and 15%, is improved 2.53%, 6.18%, and 3.11%, respectively. This is achieved by comparing with the without the introduction of the transfer strategy method, which indicates that the model-based transfer strategy proposed in this paper has a certain effect on the improvement of the classification accuracy of the “small sample” dataset, which, in turn, verifies the effectiveness of the transfer strategy.

**Figure 5:**
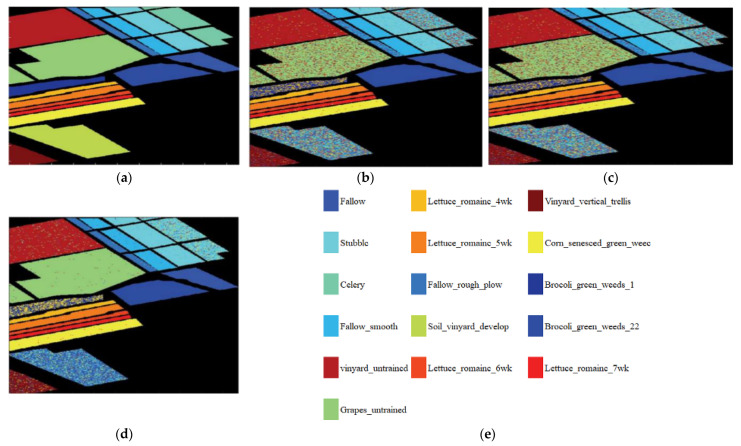
(**a**) The original images; (**b**) the CAE classification effect; (**c**) the SCAE classification; (**d**) the SCAE-MT classification effect; and (**e**) the 16 class labels.

In addition, in order to verify the classification performance of the classification method based on the SCAE-MT on the “small sample” dataset. The classification method based on the convolutional autoencoder network (CAE); the classification method based on stacking convolutional autoencoder network (SCAE); and the classification method based on optimization of the stacking convolutional autoencoder network model transfer (SCAE-MT) is calculated. The overall classification accuracy (OA) of the method at 5%, 10%, and 15% of the training set, as well as the overall accuracy (OA) of each method under the three proportions of the training samples are shown in Table 5. In regard to the Salinas dataset, the classification effect of each method under a 15% proportion of training samples is shown in Figure 5. Figure 5a–d are the original classification effects map, the classification effects map of the CAE method, the classification effects map of the SCAE method, and the classification effect map of the SCAE-MT method, respectively.

As can be seen in Table 5 and Figure 5, the SCAE-MT has an average improvement of 2.71%, 3.33%, and 3.07% in terms of overall accuracy (OA) when compared with the CAE and the SCAE methods under the training datasets of 5%, 10%, and 15%, respectively. The SCAE-MT method is significantly better than its comparative methods, which show a good classification performance. The transfer learning strategy can improve the classification accuracy of the HRSI. From the experimental results of the CAE and SCAE methods, it can be seen that the overall accuracy (OA) of the SCAE is improved by 2.43%, 1.7%, and 1.29% under the training data sets of 5%, 10%, and 15%, respectively. In regard to the deep neural network structure that stacks the convolutional autoencoder (CAE) network in a stacking manner, it can be seen that it exerts its advantages of a layer-by-layer feature extraction, indicating that each layer of the SCAE method cannot simply serve as a convolutional layer during training. The self-encoding (CAE) network is trained separately, and the small-size feature space following the upper-layer feature mapping participates in the training of the next layer, which results in it performing better in regard to classification. This, therefore, shows it has a higher classification accuracy than the CAE method. A better overall accuracy (OA) for the SCAE and CAE methods under a 5% training set can been to be 0.79% higher than that under 10% training sample set, which is 1.14% higher than that under 15% training sample set. Therefore, the experiment results show that the SCAE-MT model has a certain effect on the improvement of the classification performance of “small sample” data.

## 4. Conclusions and Prospect

In this paper, a new stacked convolutional autoencoder network model transfer (SCAE-MT) is developed in order to realize a HRSI classification method that can better solve the problems of insufficient image feature extraction, can produce large scale model training parameters, and can also more easily produce the “overfitting” and “small sample” features of HRSI data. The comparative experiments show that the overall classification accuracy of the target data set after the introduced transfer strategy is 3.94% higher than that without the transfer strategy when compared with those under a different proportion of training sample sets. The overall classification accuracy (OA) of the target domain data under the 5%, 10%, and 15% training sets is improved by 1.79% on average by organizing two convolutional self-encoders in a trestle manner and through the use of simple convolutional self-encoders. It can be seen that the trestle convolutional self-encoder used in the study can effectively extract the features of the HRSI. As a machine learning method that uses and adjusts the knowledge and model of the source domain in order to help solve the problem of the target domain, transfer learning can solve the problem of “small samples” in the target domain due to the lack of training data.

However, the proposed classification method is based on the premise that the labeled samples of the source domain dataset are sufficient. In future works, we will further study how to improve the classification accuracy and feature recognition ability of the HRSI by the utilization of a transfer learning strategy.

## Figures and Tables

**Table 1:** Basic information of the Indian Pines and the Salinas datasets.

Data	Indian Pines	Salinas
Collection location	Indiana, USA	California, U.S.
Collection equipment	AVIRIS	AVIRIS
Spectral coverage (um)	0.4∼2.5	0.4∼2.5
Data size (pixel)	145 × 145	512 × 217
Spatial resolution (m)	20	3.7
Number of bands	220	224
Number of bands after denoising	200	204
Sample size	10,249	54,129
Number of categories	16	16

**Table 2:** The 16 types of ground features in Indian Pines and Salinas datasets.

	Indian Pines		Salinas	
Category	Class Name	Number of Samples	Class Name	Number of Samples
1	Alfalfa	46	Brocoli_green_weeds_1	2009
2	Corn-notill	1428	Brocoli_green_weeds_22	3726
3	Corn-min	830	Fallow	1976
4	Corn	237	Fallow_rough_plow	1394
5	Grass-pasture	483	Fallow_smooth	2678
6	Grass-trees	730	Stubble	3959
7	Grass-pastue-mowed	28	Celery	3579
8	Hay-windrowed	478	Grapes_untrained	11,271
9	Oats	20	Soil_vinyard_develop	6203
10	Soybean-notill	972	Corn_senesced_green_weec	3278
11	Soybean-min	2455	Lettuce_romainc_4wk	1068
12	Soybean-clean	593	Lettuce_romainc_5wk	1927
13	Wheat	205	Lettuce_romainc_6wk	916
14	Woods	1265	Lettuce_romainc_7wk	1070
15	Bldg-Grass-Tree-Drives	386	Vinyard_untraincd	7268
16	Stone-Steel-Towers	93	Vinyard_vertical_trellis	1870

**Table 3:** Effects of different convolutional self-encoders on classification accuracy.

	Training Set (40%)			Training Set (80%)		
Number	1	2	3	1	2	3
OA (%)	90.67	95.65	95.82	93.32	97.03	97.17

**Table 4:** Overall classification accuracy under different proportions of training sample sets.

**Index**	**Method**	**Salinas Dataset**		
		**5%**	**10%**	**15%**
	No transfer strategy	80.58	82.74	87.13
OA (%)	Introduction of transfer strategy	**83.11**	**88.92**	**90.24**

**Table 5:** The overall classification accuracy under different proportions of sample sets.

Method	CAE (OA%)	SCAE (OA%)	SCAE-MT (OA%)
5%	79.19	81.62	**83.11**
10%	84.77	86.41	**88.92**
15%	86.53	87.82	**90.24**

## Data Availability

Not applicable.
